# Supplementary material for: Decoding Functional High-Density Lipoprotein Particle Surfaceome Interactions
Source: Int J Mol Sci. 2022 Aug 22;23(16):9506. doi: 10.3390/ijms23169506 (PMC9409371; doi:10.3390/ijms23169506)
Supplement: Supplementary file 1 [file ijms-23-09506-s001.zip › ijms-1852166-supplementary-Figures.pdf]

# Supplemental Materials

## Decoding Functional High-Density Lipoprotein Particle Surfaceome Interactions

Kathrin Frey [1,2,†], Sandra Goetze [1,3,4,*,†], Lucia Rohrer [2], Arnold von Eckardstein [2] and Bernd Wollscheid [1,3,*]

[1] Institute of Translational Medicine, Department of Health Sciences and Technology, ETH Zurich, 8093 Zurich, Switzerland

[2] Institute for Clinical Chemistry, University Hospital Zurich, 8091 Zurich, Switzerland

[3] Swiss Institute of Bioinformatics (SIB), 1015 Lausanne, Switzerland

[4] ETH PHRT Swiss Multi-Omics Center (SMOC), 8093 Zurich, Switzerland

* Correspondence: sandra.goetze@hest.ethz.ch (S.G.); bernd.wollscheid@hest.ethz.ch (B.W.); Tel: +41-44-633-3001 (S.G.)

† These authors contributed equally to this work.

# Supplementary Tables:

**Table S1**. List of identified peptides in auto-CSC experiments with EA.hy926 cells, HAECs, HEPG2 cells, THP1 cells, activated THP1 cells, and foam cells. Identifications are filtered for FDR < 0.01 and NxS/T motifs.

**Table S2**. Matrix of all identified proteins in auto-CSC experiments with EA.hy926 cells, HAECs, HEPG2 cells, THP1 cells, activated THP1 cells, and foam cells.

**Table S3**. List of ranked and scaled protein abundances and the peptide counts (used for quantification) captured in auto-CSC experiments with EA.hy926 cells, HAECs, HEPG2 cells, THP1 cells, activated THP1 cells, and foam cells.

**Table S4**. Safequant output of the auto-CSC experiments quantitatively comparing VEGF-A treated vs untreated HAEC surfaceomes.

**Table S5**. Statistics from the Gene Ontology analysis of genes regulated by VEGF-A in HAECs.

**Table S6**. Gene Ontology protein annotation of the analysis of VEGF-A-sensitive proteins on HAECs.

**Table S7**. Protein content of rHDL as identified by COMET. Peptide and protein identifications were filtered with an FDR of 1%. Common contaminants and keratins were removed from the protein list.

**Table S8**. Safequant output of the HATRIC-LRC experiment with APOA1, lipidated APOA1, and HDL as ligands on EA.hy926 cells.

## Supplementary Figures:

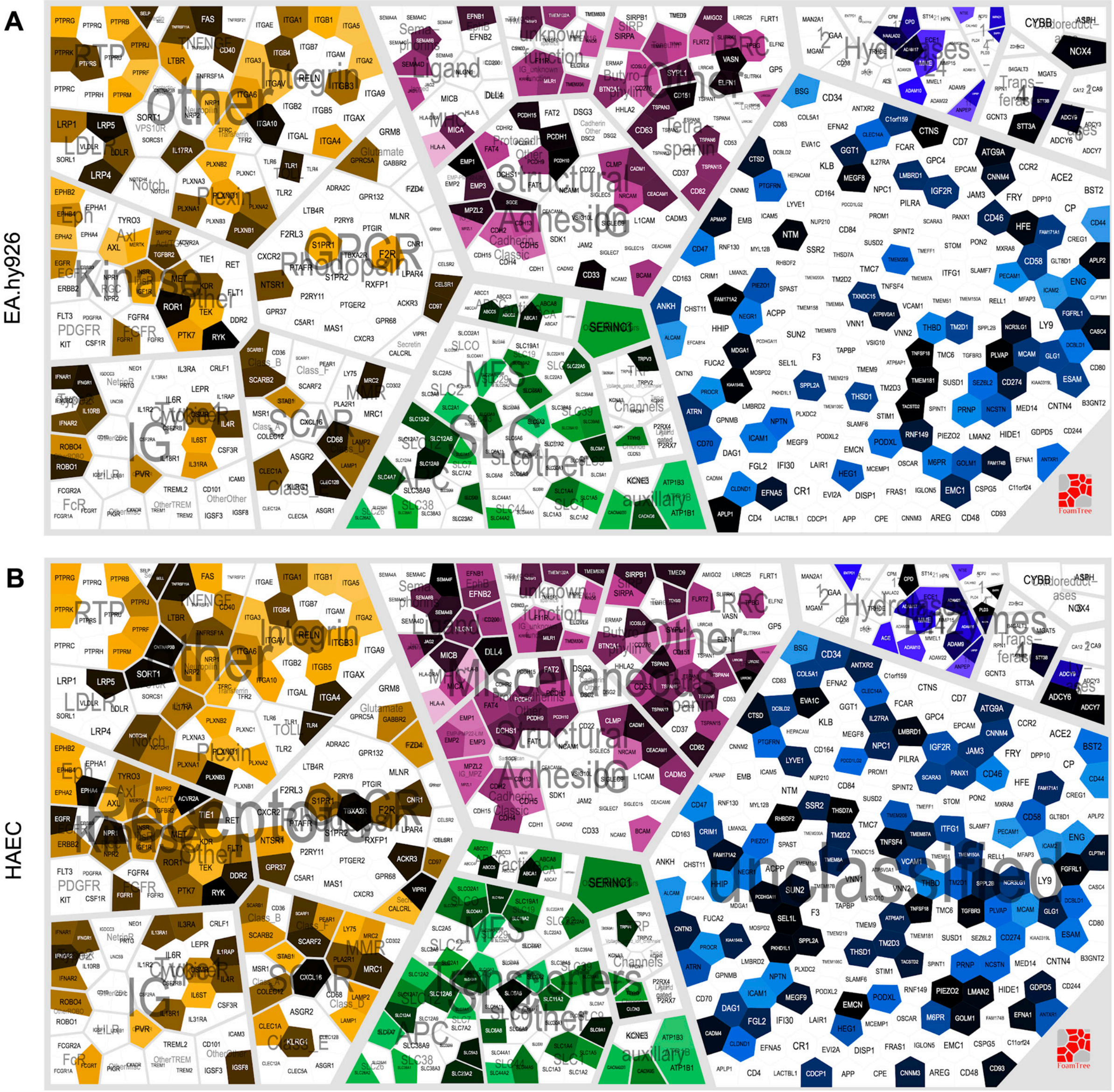

C

HEPG2

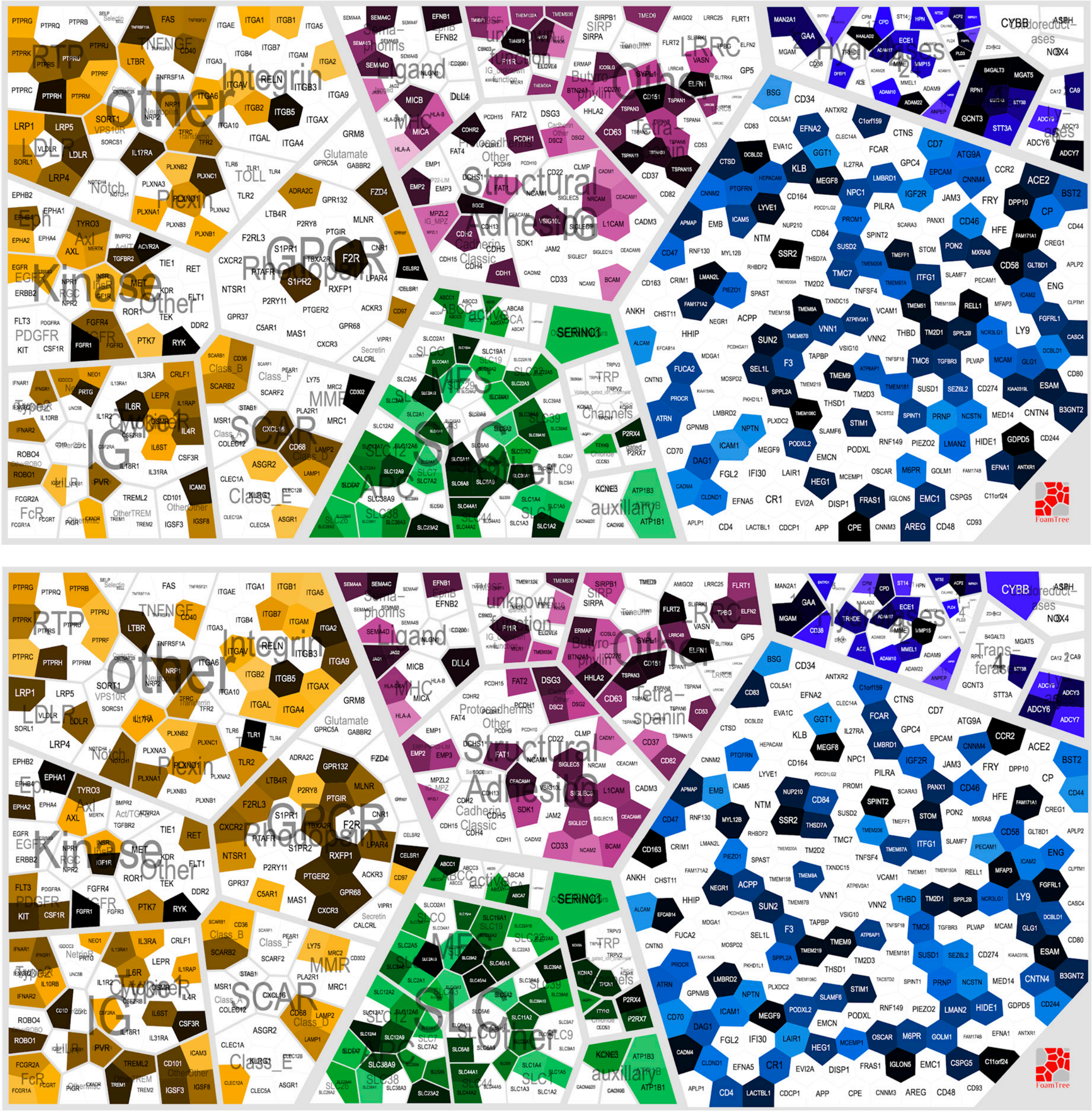


D

THP1

**E** activated THP1

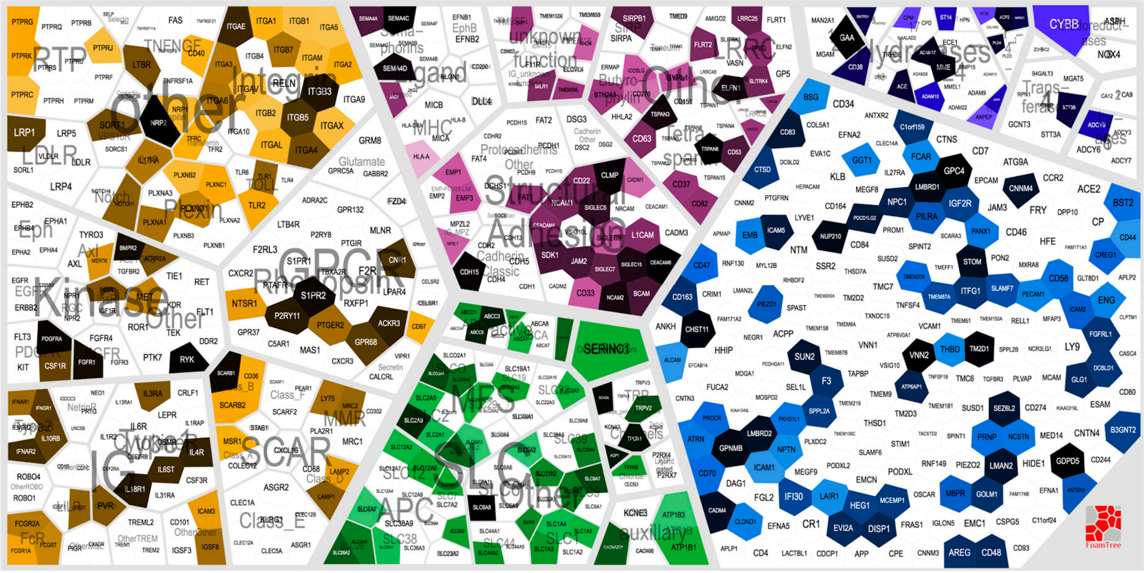

**F** Foam cells

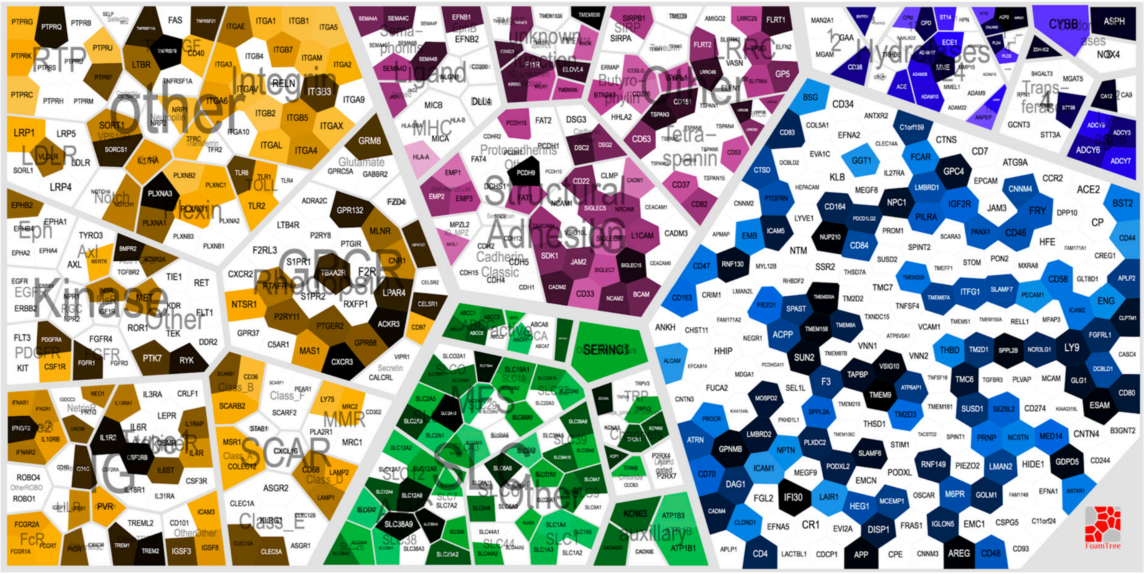

**Figure S1.** Voronoi treemaps of the quantitative surfaceome of different cell lines: (**A**) EA.hy926 cells, (**B**) HAECs, (**C**) HEPG2 cells, (**D**) THP1 cells, (**E**) activated THP1 cells, and (**F**) foam cells. The background proteome comprises all identified proteins across the different cell types. Color scale corresponds to the cell type-specific ranked and scaled abundance levels of the proteins.

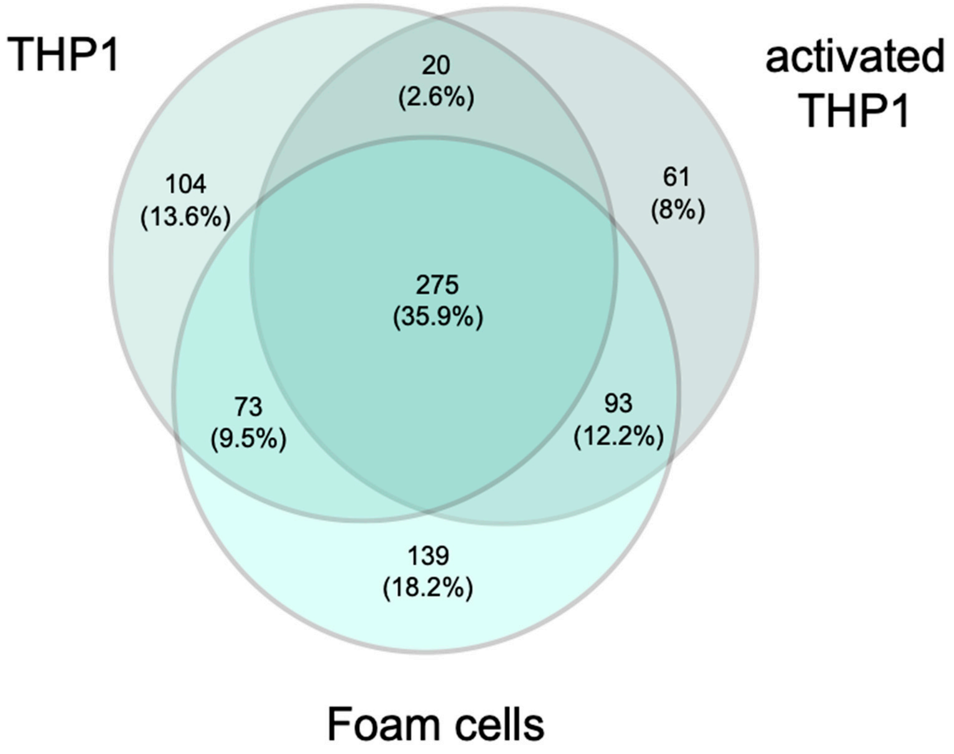


**Figure S2**. Venn diagram of overlap of identified proteins in the auto-CSC experiments with THP1 cells, activated THP1 cells, and foam cells.

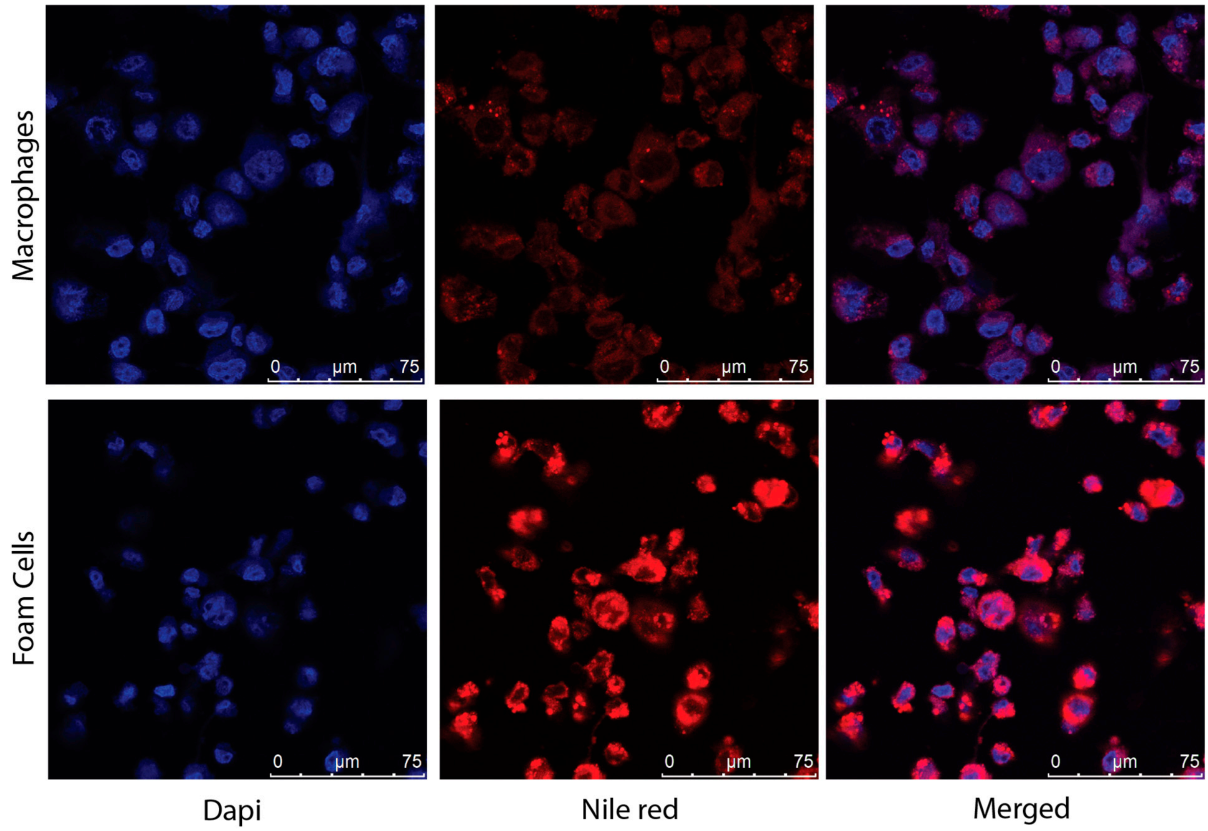


**Figure S3.** Microscopy images of the lipid content within THP1 cells, activated THP1 cells, and foam cells. Lipids were stained with Nile red.

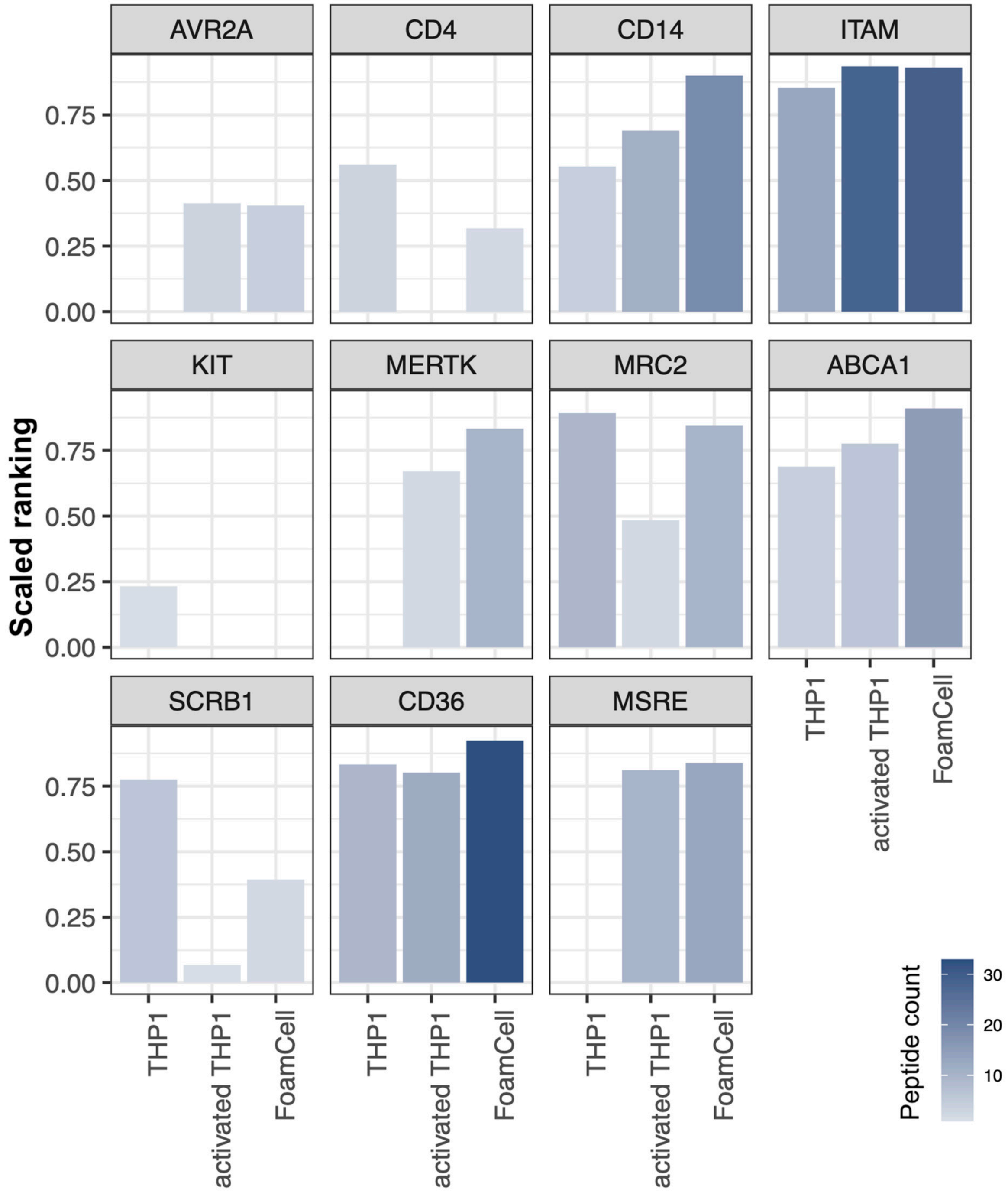


**Figure S4**. Bar charts of selected proteins and their scaled ranked abundances determined with auto-CSC on THP1 cells, activated THP1 cells, and foam cells. Included are proteins previously shown to have altered surface abundance upon THP1 differentiation [1]: AVR2A, CD4, CD14, ITAM, KIT, MERTK, and MRC2. Data for the APOA1/HDL receptors ABCA1 and SCRB1 as well as the oxLDL receptors CD36 and MSRE [2] are also shown.

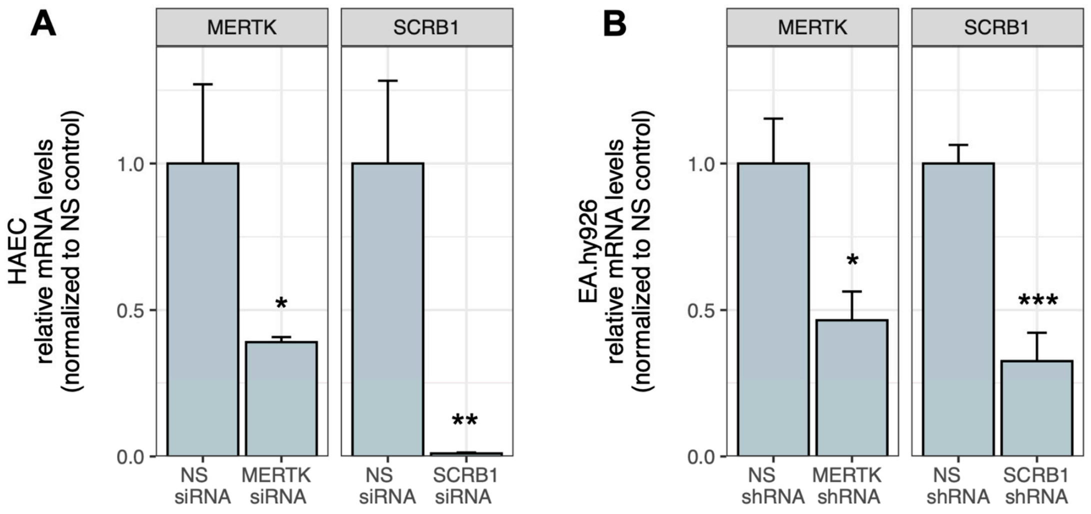


**Figure S5.** mRNA levels upon silencing of *MERTK* and *SCRB1* in EA.hy926 and HAECs. Significance of *MERTK* and *SCRB1* silencing using shRNA in EA.hy926 cells and siRNA in HAECs compared to control cells treated with non-silencing (NS) vector or control, respectively, was assessed with Student's *t*-test (* $p < 0.05$, ** $p < 0.01$, *** $p < 0.001$).

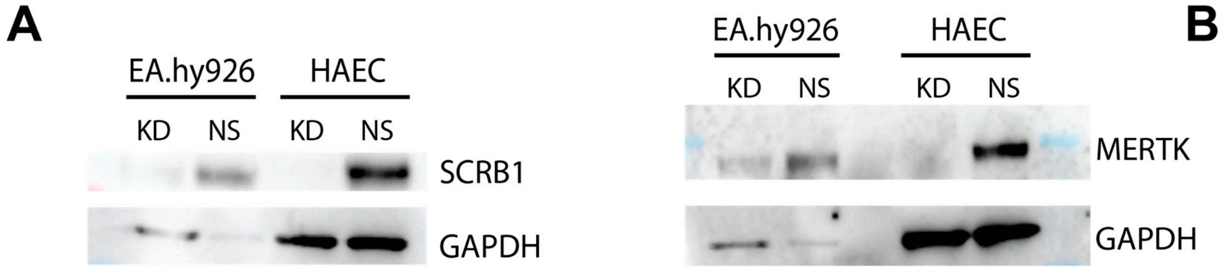


**Figure S6.** Western blot analysis of total protein abundance levels upon silencing of (**A**) *SCRB1* and (**B**) *MERTK* in EA.hy926 cells and HAEC with shRNA or siRNA, respectively (KD), and upon treatment with non-silencing controls (NS).

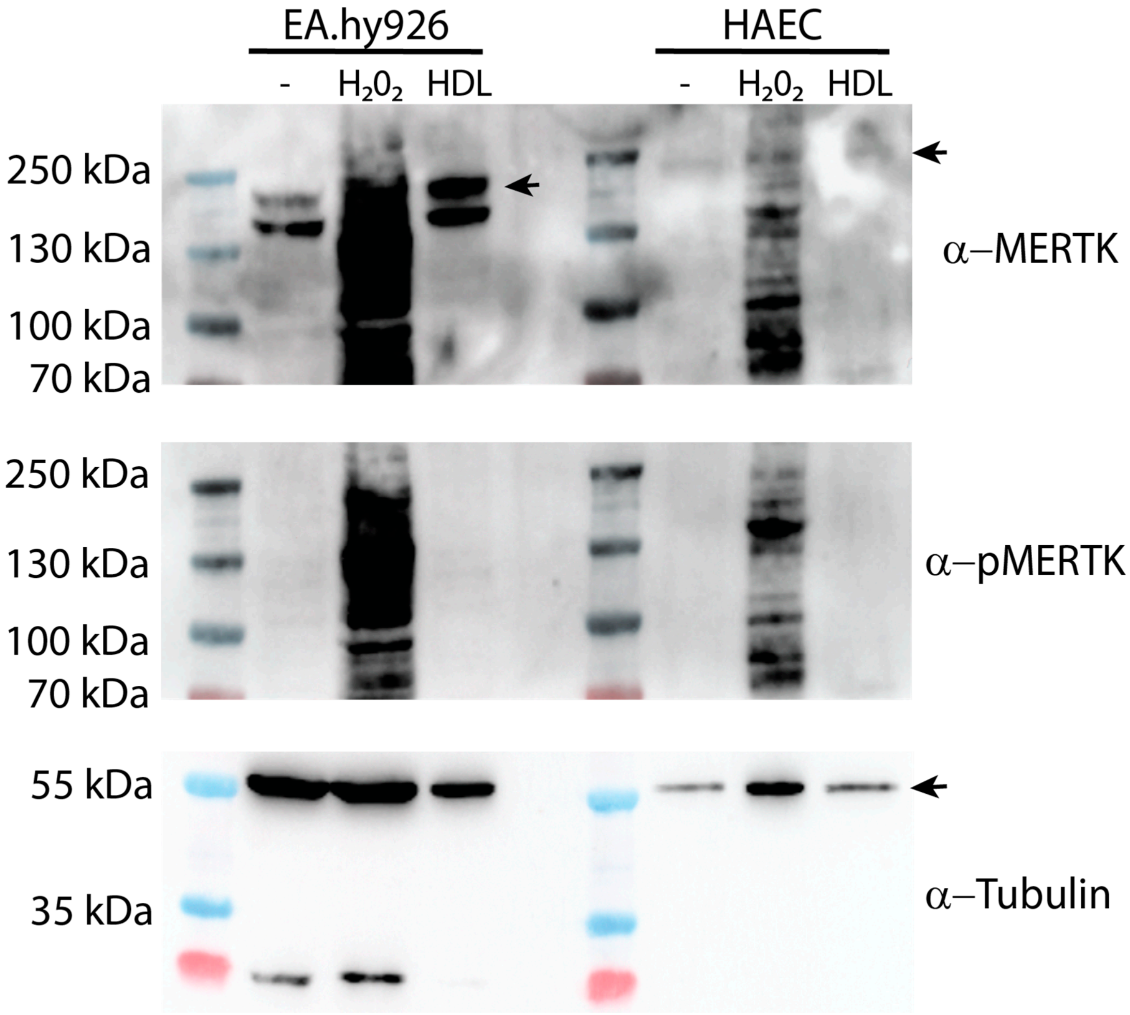


**Figure S7.** Effect of HDL treatment on phosphorylation of MERTK in EA.hy926 cells and HAECs as shown by Western blot. $H_2O_2$ treatment served as positive control [3]. Tubulin was used as loading control.

# References:


1. Kalxdorf, M.; Gade, S.; Christian Eberl, H.; Bantscheff, M. Monitoring Cell-surfaceN-Glycoproteome Dynamics by Quantitative Proteomics Reveals Mechanistic Insights into Macrophage Differentiation. *Molecular & Cellular Proteomics* 2017, *16*, 770–785.
2. Das, R.; Ganapathy, S.; Mahabeleshwar, G.H.; Drumm, C.; Febbraio, M.; Jain, M.K.; Plow, E.F. Macrophage Gene Expression and Foam Cell Formation Are Regulated by Plasminogen. *Circulation* **2013**, *127*, 1209–1218, e1–e16.
3. Anwar, A.; Keating, A.K.; Joung, D.; Sather, S.; Kim, G.K.; Sawczyn, K.K.; Brandão, L.; Henson, P.M.; Graham, D.K. Mer Tyrosine Kinase (MerTK) Promotes Macrophage Survival Following Exposure to Oxidative Stress. *J. Leukoc. Biol.* **2009**, *86*, 73–79.
